# Comment on Covelli et al. Extracorporeal Shock Wave Therapy (ESWT) vs. Exercise in Thumb Osteoarthritis (SWEX-TO): Prospective Clinical Trial at 6 Months. *Life* 2024, *14*, 1453

**DOI:** 10.3390/life15030384

**Published:** 2025-02-28

**Authors:** Feray Karademir, Tüzün Fírat

**Affiliations:** Hacettepe University, Faculty of Physical Therapy and Rehabilitation, Department of Hand Surgery and Rehabilitation, 06100 Ankara, Turkey; tuzun@hacettepe.edu.tr

We read the study by Covelli et al. [1] with great interest. We appreciate the authors for their contribution. However, we would like to address some methodological concerns that we believe warrant attention.

The primary aim of this study, as stated in the article, is to compare the effects of Extracorporeal Shock Wave Therapy (ESWT) versus exercise in patients with trapeziometacarpal osteoarthritis (TMC OA). However, the statistical approach used to analyze the results appears misaligned with the aim of the study. Specifically, the study used a four-way repeated-measures Wilcoxon test for each group, which does not allow for an appropriate comparison of treatment efficacy between the two modalities. Given that two different interventions (ESWT and exercise) were being compared, a two-way repeated-measures ANOVA would have been a more appropriate statistical method to accurately assess treatment effectiveness and time by treatment interactions. Furthermore, the regression analyses provided (both multivariable and univariable) do not appear to clarify or address the comparisons of treatment effects between the two modalities. It is essential that statistical analyses accurately reflect the study design and aim to avoid generating misleading conclusions.

Another issue of concern is the use of ultrasound to guide the application of ESWT. The article mentions that the probe used for ESWT was large in size and that the trapeziometacarpal (TMC) joint was identified using ultrasound to guide the therapy. However, the TMC joint is not particularly difficult to palpate or anatomically identify, which raises questions about the need for ultrasound in this study. The rationale for using ultrasound remains unclear, and it is important to justify the choice of this technique. If the goal was to ensure precision in targeting the treatment area, the authors should have provided further explanation of the specific advantages of using ultrasound over traditional palpation methods. Additionally, if precision was indeed the primary concern, one would expect a more detailed discussion of the ESWT application technique itself, including its indications and how it was optimized for TMC OA. 

Finally, the Discussion section does not explain why ESWT was specifically chosen as a treatment modality for TMC OA. While the authors briefly mention the general mechanism of ESWT in terms of pain inhibition, the article does not adequately address the unique pathophysiology of TMC OA. The mechanism of action of ESWT in TMC OA has not been sufficiently investigated, resulting in a gap in understanding the rationale behind the preference for this treatment over other alternatives. A more focused discussion on how ESWT interacts with the specific biological processes involved in TMC OA, such as cartilage degradation, osteophyte formation, or joint instability, would provide greater insight into the relevance of this treatment choice.

In conclusion, while this study offers valuable insights into the comparison of ESWT and exercise therapy for TMC OA, we hope that the aforementioned issues will be addressed in future revisions or discussions, since doing so would enhance the overall validity and impact of the findings.

## References

[1] Covelli I., De Giorgi S., Di Lorenzo A., Pavone A., Salvato F., Rifino F., Moretti B., Solarino G., Notarnicola A. (2024). Extracorporeal Shock Wave Therapy (ESWT) vs. Exercise in Thumb Osteoarthritis (SWEX-TO): Prospective Clinical Trial at 6 Months. Life.

